# The FAANG Data Portal: Global, Open-Access, “FAIR”, and Richly Validated Genotype to Phenotype Data for High-Quality Functional Annotation of Animal Genomes

**DOI:** 10.3389/fgene.2021.639238

**Published:** 2021-06-17

**Authors:** Peter W. Harrison, Alexey Sokolov, Akshatha Nayak, Jun Fan, Daniel Zerbino, Guy Cochrane, Paul Flicek

**Affiliations:** European Molecular Biology Laboratory, European Bioinformatics Institute, Cambridge, United Kingdom

**Keywords:** FAANG, functional annotation, phenotype to genotype, FAIR data, agricultural genomics, Data Portal, open access, metadata validation

## Abstract

The Functional Annotation of ANimal Genomes (FAANG) project is a worldwide coordinated action creating high-quality functional annotation of farmed and companion animal genomes. The generation of a rich genome-to-phenome resource and supporting informatic infrastructure advances the scope of comparative genomics and furthers the understanding of functional elements. The project also provides terrestrial and aquatic animal agriculture community powerful resources for supporting improvements to farmed animal production, disease resistance, and genetic diversity. The FAANG Data Portal (https://data.faang.org) ensures Findable, Accessible, Interoperable and Reusable (FAIR) open access to the wealth of sample, sequencing, and analysis data produced by an ever-growing number of FAANG consortia. It is developed and maintained by the FAANG Data Coordination Centre (DCC) at the European Molecular Biology Laboratory's European Bioinformatics Institute (EMBL-EBI). FAANG projects produce a standardised set of multi-omic assays with resulting data placed into a range of specialised open data archives. To ensure this data is easily findable and accessible by the community, the portal automatically identifies and collates all submitted FAANG data into a single easily searchable resource. The Data Portal supports direct download from the multiple underlying archives to enable seamless access to all FAANG data from within the portal itself. The portal provides a range of predefined filters, powerful predictive search, and a catalogue of sampling and analysis protocols and automatically identifies publications associated with any dataset. To ensure all FAANG data submissions are high-quality, the portal includes powerful contextual metadata validation and data submissions brokering to the underlying EMBL-EBI archives. The portal will incorporate extensive new technical infrastructure to effectively deliver and standardise FAANG's shift to single-cellomics, cell atlases, pangenomes, and novel phenotypic prediction models. The Data Portal plays a key role for FAANG by supporting high-quality functional annotation of animal genomes, through open FAIR sharing of data, complete with standardised rich metadata. Future Data Portal features developed by the DCC will support new technological developments for continued improvement for FAANG projects.

## Introduction

The Functional Annotation of Animal Genomes Project (FAANG) is a coordinated action to improve availability of high-quality functional annotation of farmed and companion animal genomes (Andersson et al., 2015; Tuggle et al., 2016; Giuffra et al., 2019; Clark et al., 2020). Rich genome-to-phenome resources are of particular importance in domesticated animals of commercial importance for efforts to increase agricultural production, but the available resources also impact upon the fundamental understanding of functional elements, biomedical science, evolution, and the environment. The FAANG project comprises multiple globally distributed consortia working across a growing range of species and committed to high-quality data production and interpretation. The FAANG Data Coordination Centre (DCC) at the European Molecular Biology Laboratory's European Bioinformatics Institute (EMBL-EBI) ensures that all data generated by the project is richly described, consistently reported, openly available, reusable, and clearly presented (Harrison et al., 2018). The FAANG Data Portal^1^ plays a pivotal role by coordinating and presenting the wealth of data generated by the project to the scientific community. Its primary purpose is to provide a searchable, unified view of the multi-omic FAANG data held across specialised EMBL-EBI archives. Its web interface and Application Programming Interface (API) supports the identification and download of FAANG generated and associated community datasets, as well as exploring the associated rich validated metadata, protocols, and publications. Here we describe the key features of this rich genome to phenome resource and look to future developments that will expand it as research advances.

## Methods

**Figure 1:**
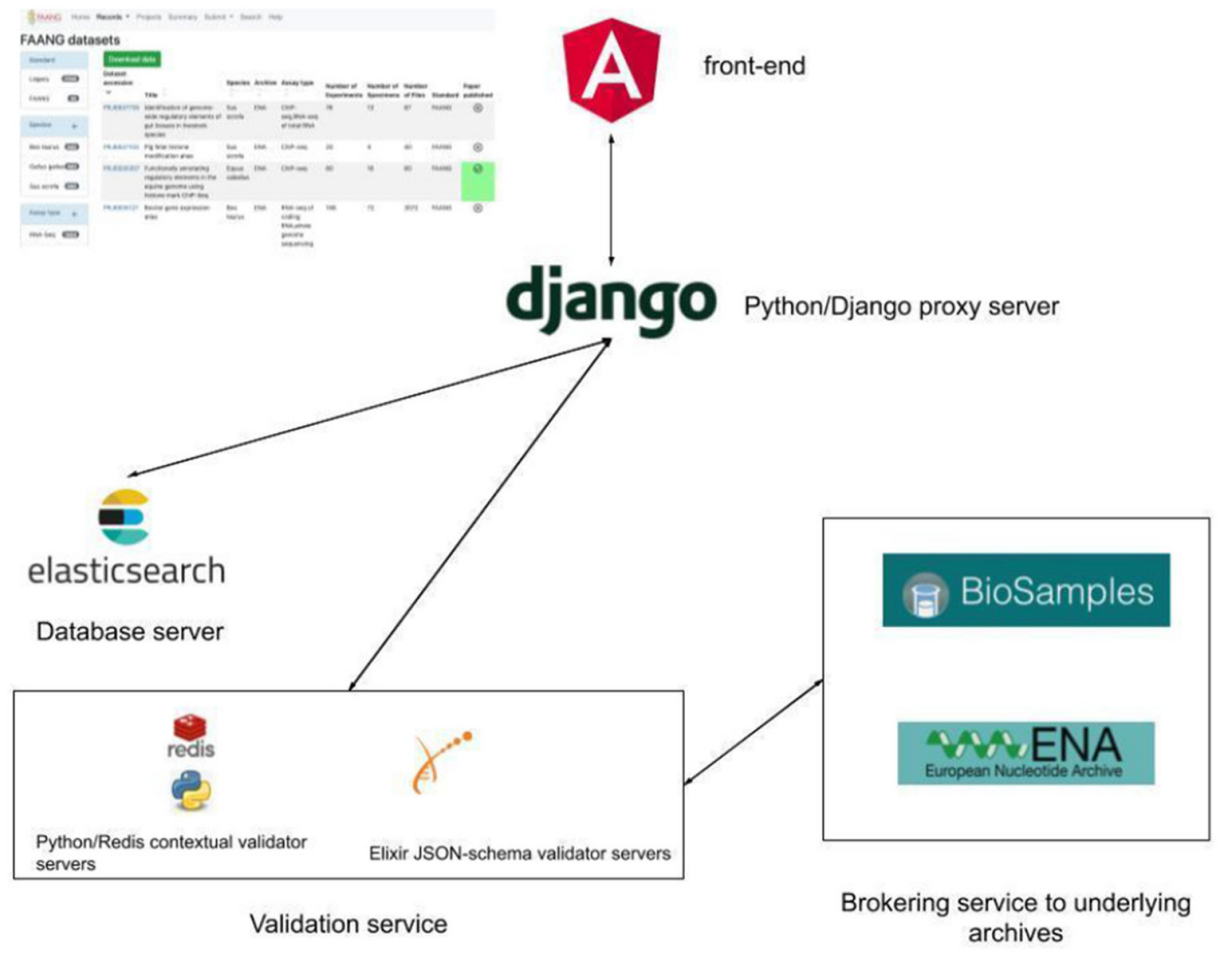
FAANG Data Portal architecture with local Elasticsearch metadata database, python, and JSON-schema contextual validation and brokering of validated data to underlying public archives.

The FAANG Data Portal^1^ comprises a modern technology stack with a microservice architecture design. Establishing each component as a separate microservice enables more flexible, scalable, and faster maintenance and development of new features. The front-end Data Portal is written in Angular, an open-source web application framework. Continuous integrated deployment is managed by Kubernetes, an open-source system to manage the Docker containers which contain all of the code and software required to launch and run the Data Portal components (Figure 1). It is deployed on the EMBL-EBI Embassy Cloud infrastructure^2^, enabling a flexible and efficient use of computational resources. The selection of open-source frameworks matches the FAANG projects and EMBL-EBI's ethos of open development. The code has permissive Apache 2.0 licencing to allow the community to reuse and benefit from any of the codebase. The use of Kubernetes and Docker containers would permit the Data Portal to be easily deployed onto other cloud infrastructures, such as Google Cloud or Amazon Web Services, if required, aiding long-term sustainability. Another advantage of deployment on the EMBL-EBI Embassy Cloud is that the underlying datasets are held in the same data centres for rapid access. Metadata search, from both the Data Portal and programmatic API, is supported by a local backend Elasticsearch metadata database. The Elasticsearch database is essentially a customised full-text search engine built explicitly for indexing the FAANG metadata and supports partial word search (through custom tokenisation) with high specificity. Rather than hosting duplicate copies of the data, the underlying data files remain located in the specialised data archives such as the European Nucleotide Archive (Amid et al., 2020) and European Variation Archive. The Data Portal includes direct links and bulk download support to directly obtain the relevant data files. It also supports its own FTP site for hosting protocol and presentation files not suitable for archive submission including track hub files. Track data hubs provide a mechanism to store third-party genome annotations within specifically formatted files for distribution and display. Track hub file formats are optimised for display through web-based genome browsers. FAANG utilises the Track Hub Registry^3^ to seamlessly enable FAANG community generated trackhub annotations to be made discoverable for use with the UCSC (Lee et al., 2020) and Ensembl Genome Browsers (Yates et al., 2020). The Data Portal infrastructure is already in place to support track hub submissions, utilising the FAANG Data Portal FTP site and EMBL-EBI Track Hub Registry (https://trackhubregistry.org/). Presentation and linking to FAANG track hubs will be improved in future releases of the Data Portal, so that track hubs are clearly associated with FAANG datasets and users can more quickly view them in the UCSC (Lee et al., 2020) and Ensembl Genome Browsers (Yates et al., 2020).

Metadata standards are held in GitHub in JSON schema for ease of rendering on the Data Portal and use in the validation system. This also allows anyone in the community to propose a metadata change through a pull request. The validation and submission brokering data processing is performed in python with asynchronous data processing also hosted in an Embassy Cloud instance with user interaction through the FAANG Data Portal interface. Documentation is managed through a readthedocs GitHub instance^4^ that allows updating site text without the need for redeployment of the full Data Portal.

## Results

### The FAANG Data Portal

**Figure 2:**
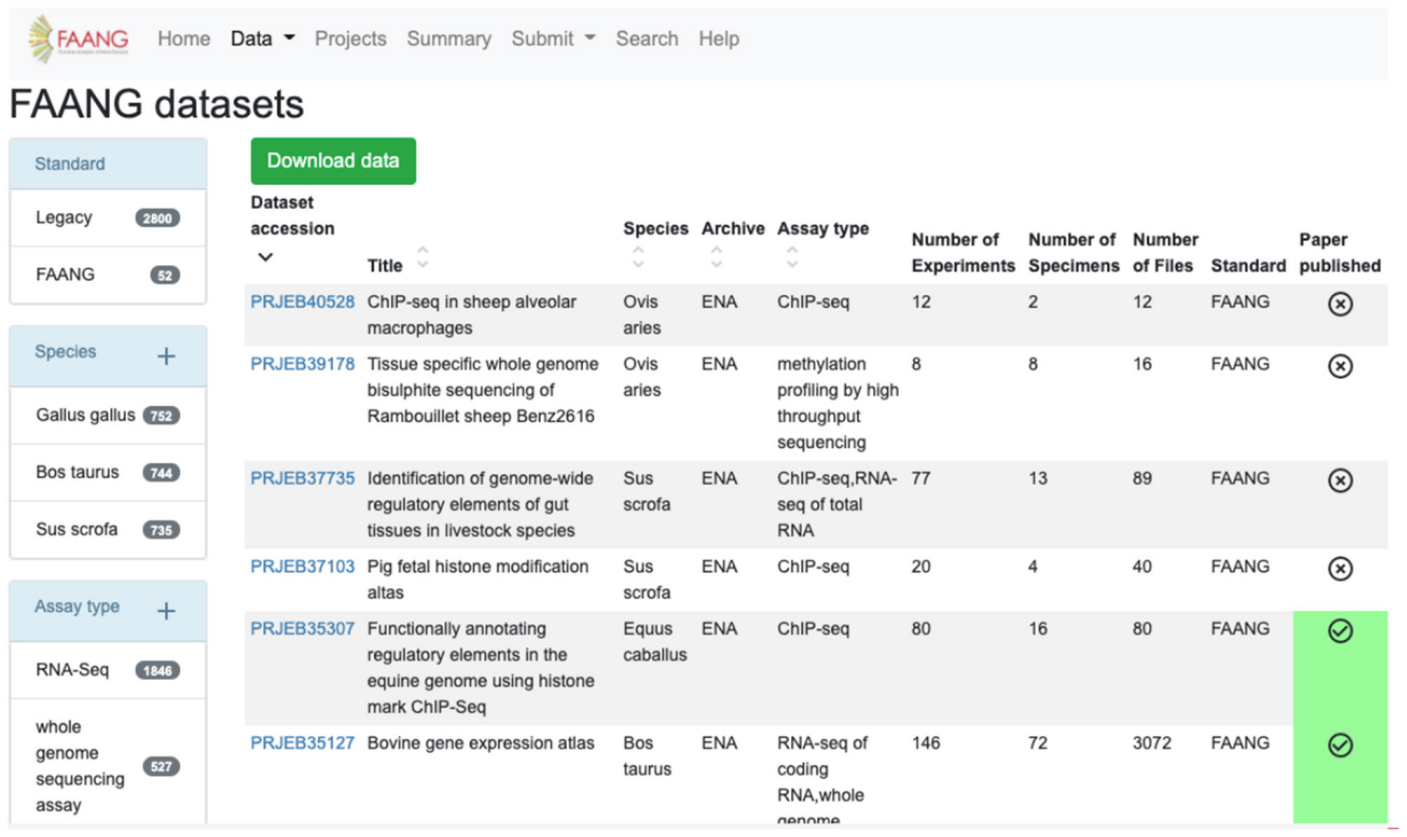
FAANG Data Portal presenting rich ‘omic datasets to the community complete with preconfigured data filters, automated literature scraping, and direct links to data files in underlying archives (https://data.faang.org/dataset).

The FAANG Data Portal's primary function is to collate and clearly present the wealth of FAANG data to the community. FAANG data is divided into clear record sections within the Data Portal comprising organism metadata BioSample records, specimen metadata BioSample records, full datasets, individual raw data files, and individual processed analysis files. The portal enables browsing of any of these interconnected sections making it possible to navigate to all of the data records that stem from a given organism. The record table views (Figure 2) provide preconfigured filtering to narrow down the search, for example, by species or assay type. The filters show the number of records in each field.

Tables are sortable by columns, and the filtered table can be downloaded into CSV or tabular format. As well as the data records, it is also possible to browse and search the extensive collection of protocols. The FAANG data portal automatically scans for publications associated with any of the record identifiers contained in the FAANG Data Portal and automatically downloads and links these publications to their associated records. Other key Data Portal features include global summary statistics, predictive text search, and detailed documentation. The site is supported by an active helpdesk backed by the EMBL-EBI FAANG DCC that supports users with data validation, submission, and retrieval of data. Additionally, the Data Portal collates relevant publicly available functional data generated outside of the FAANG consortium into comparable tables. When these data sets do not meet current stringent FAANG standards, they are clearly labelled with a Legacy tag.

**Figure 3:**
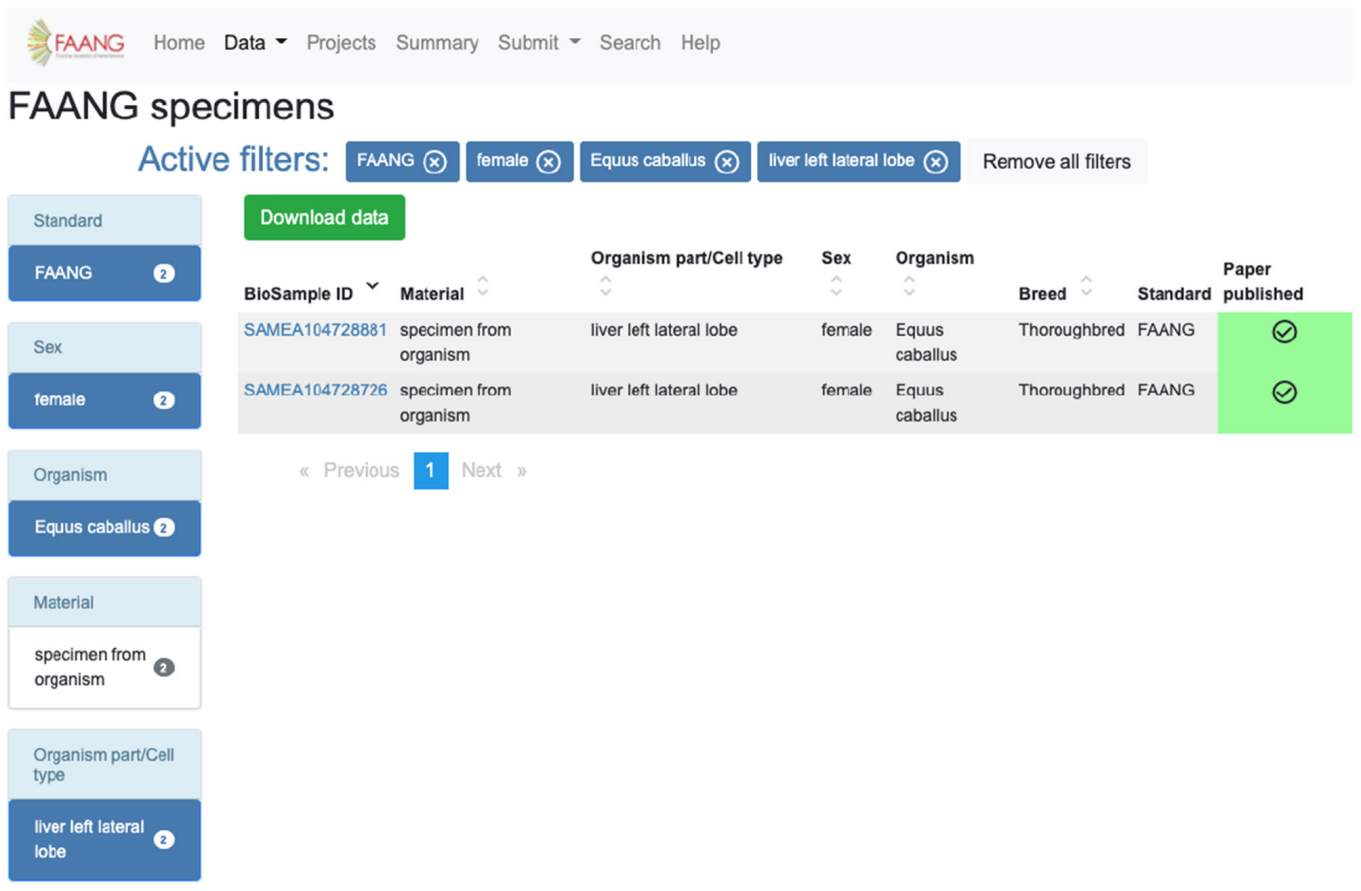
FAANG Data Portal specimen table utilising filters to obtain specimens from *Equus caballus* females from the liver left lateral lobe (https://data.faang.org/specimen?standard=FAANG&sex=female&organism=Equus%20caballus&organismpart_celltype=liver%20left%20lateral%20lobe).

The identification and acquisition of data relevant to a user's scientific interests is the main use case for the portal. The site provides extensive preconfigured filters for exploring data tables, search functionality, and an API. For example, a user interested in obtaining specimens from *Equus caballus* females from the liver left lateral lobe can utilise the preconfigured filters on the specimen data exploration table to select species, sex, and organism part and exclude legacy data (Figure 3). The same search is possible using the highly customisable API (https://dcc-documentation.readthedocs.io/en/latest/api/) to return the results programmatically. The current Data Portal search function ranks data based on keyword prioritisation, so for the above example the search would return a range of other datasets that share one or more of the keywords. In a future Data Portal release, the search page (https://data.faang.org/search) will be updated to use an advanced query language that would allow for more advanced text searches. Once records of interest have been identified, data files can be downloaded from within the data portal without having to navigate away to the underlying archives. Continuing with the above example, the 18 files that include Chip-Seq, methylation profiling, and RNA-Seq can be initiated for download from within the Data Portal from the specific specimen pages (https://data.faang.org/specimen/SAMEA104728881).

**Figure 4:**
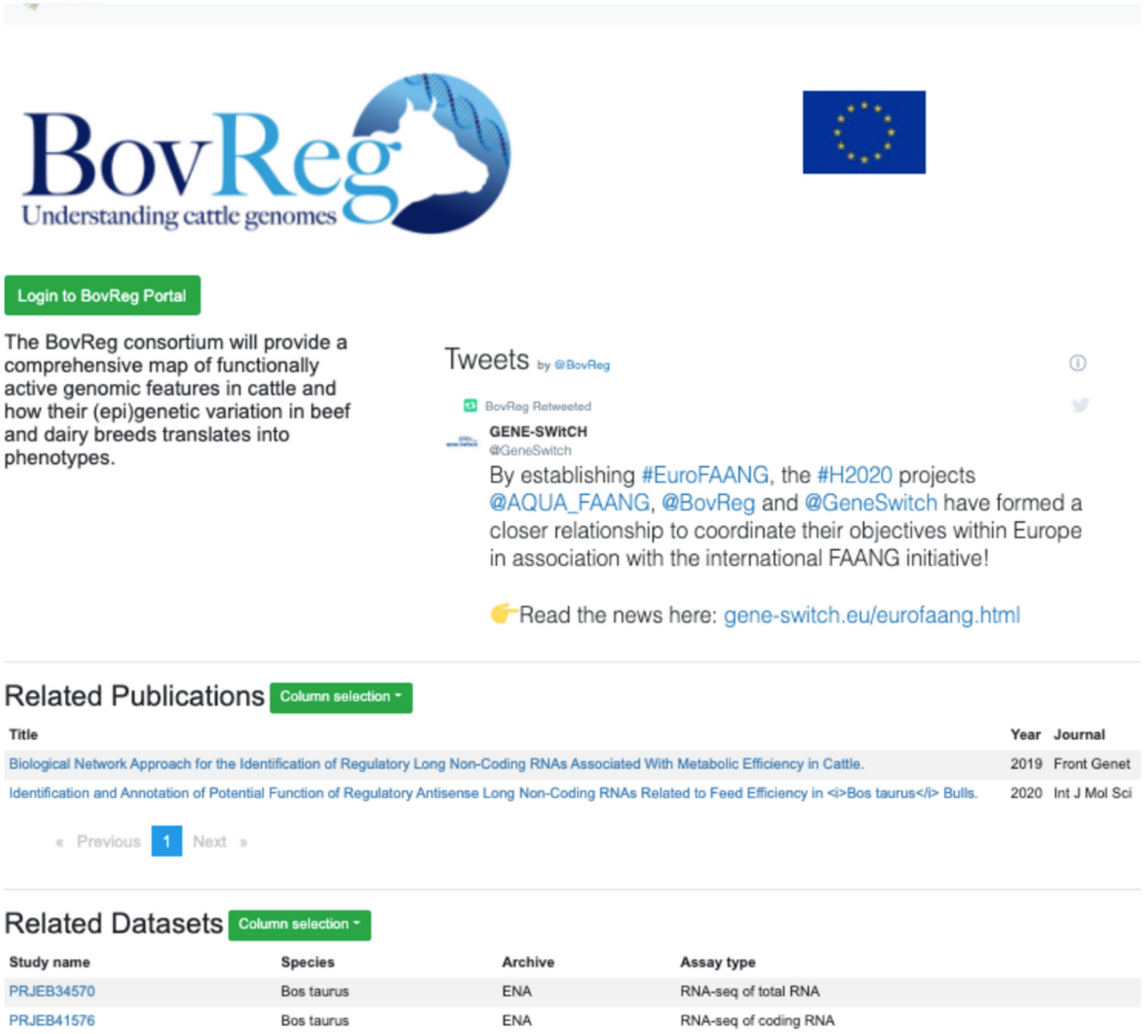
Project-specific subportal views offer the full functionality of the FAANG site pre-filtered to data from a particular consortia (https://data.faang.org/projects/BovReg).

A recent new feature is project-specific subportal views, initially developed to support the EU-funded Horizon 2020 FAANG projects GENE-SWiTCH, BovReg, and AQUA-FAANG. This feature is now available for any current or future FAANG consortia. These project-specific portals distribute the full functionality of the Data Portal with datasets pre-filtered to show only those from the specific project (Figure 4). A customised project page includes relevant information such as a social media stream and can be further tailored with features to support the project's data presentation requirements. Project specific portal pages are constructed using standardised files on GitHub^5^, allowing for new project pages to be quickly established.

### Metadata Validation and Data Submission Brokering

**Figure 5:**
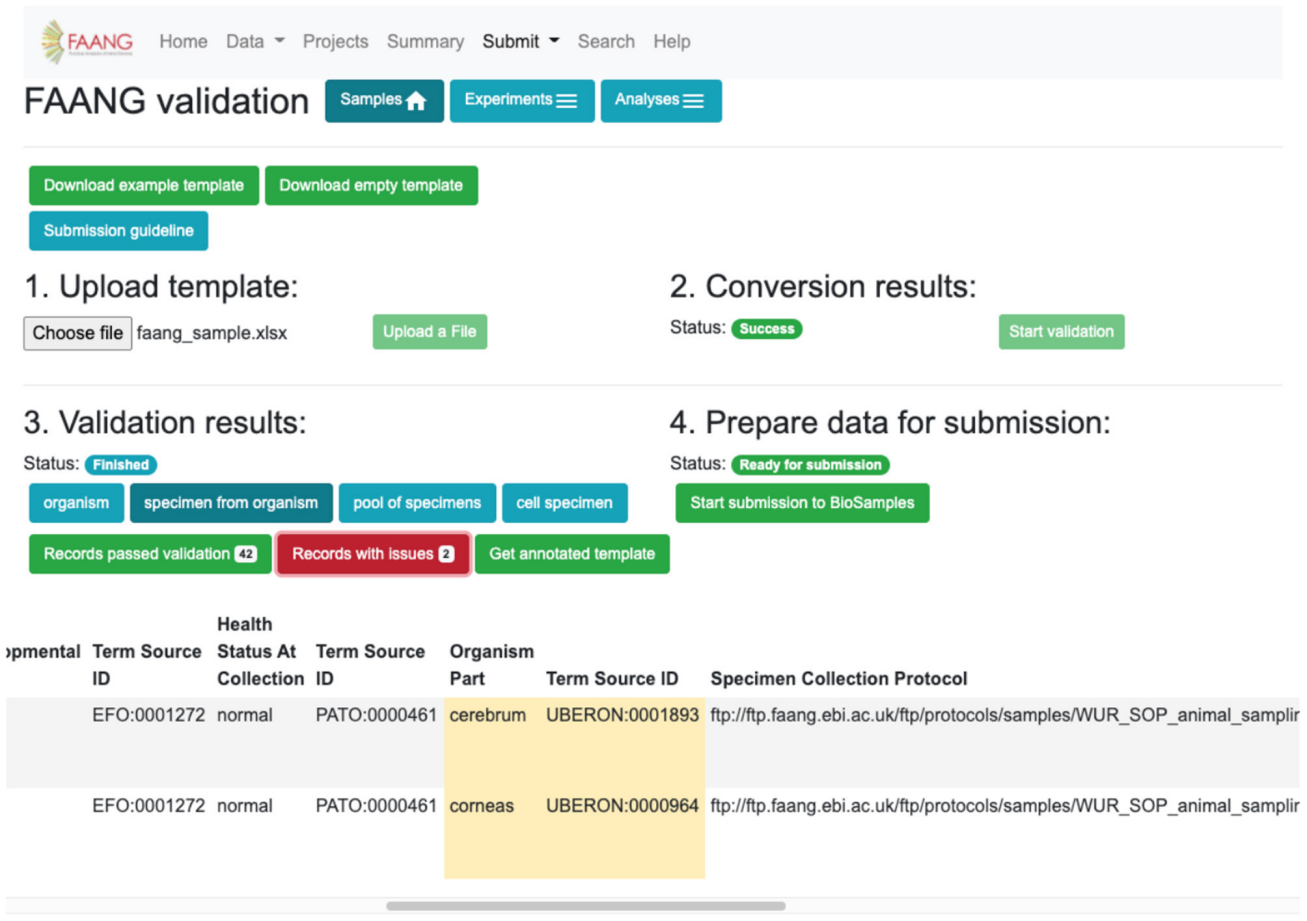
Data validation and submission brokering service flag metadata errors and improvements for correction before submission can be made (https://data.faang.org/validation/samples).

Metadata validation is fully integrated into the FAANG Data Portal with an improved web interface based on user experience testing (Figure 5). The improvements further standardise FAANG submissions, streamline the submission process, and lower the barrier to open data sharing. At the same time, we added fully brokered submissions into the underlying data archives, whereby users who supply their EMBL-EBI credentials can have submissions made on their behalf. This simplifies and accelerates the submission of FAANG data. The Data Portal includes clear guidance for using FAANG's rich metadata standards and provides an intuitive validation interface that ensures every FAANG submission meets this high standard by annotating and requiring improvements prior to submission. A metadata spreadsheet accompanies the data for each submission. This spreadsheet is validated against the FAANG standards and then used to construct the file format required for a brokered archive submission. The Data Portal will also host the corresponding protocols in its FTP directory, thus ensuring long-term availability and cross project standardisation.

The submission brokering service handles the full submission process of sample metadata to the EMBL-EBI BioSamples archive. For sequencing and analysis submissions, the submission brokering system handles the metadata registration and study creation and just requires additionally that the user uploads the sequencing or analysis files direct to the European Nucleotide Archive (ENA) FTP submission server. This ensures that the files are available when the brokering system makes the submission and can correctly associate the files with the created study. FAANG supports all of the data types and file formats currently accepted by the BioSamples and ENA archives and periodically checks for the requirement to support new technologies and file formats. The Data Portal handles any immediate submission errors and presents them back to the submitter. Errors subsequently discovered during post-submission processing by the underlying archives go straight to the submitters' registered email address. Upon a successful submission, the submitter is provided with a receipt that contains all of the assigned identifiers for their submission, which can then be referenced in their publications. Data files typically appear on the FAANG data portal within 48 h, once they have been made publicly available by the underlying archives.

### Open-Access and FAIR Data

The DCC strives to meet the highest standards of open and FAIR data recording (Wilkinson et al., 2016). All FAANG data is easily findable as it is assigned a persistent globally recognised identifier by the EMBL-EBI archives, to which the Data Portal brokers submitted datasets. The FAANG metadata standards and associated validation tooling ensures that all data has associated rich metadata, as it holds submission until all standards are appropriately met. The data records are easily found on the Data Portal using any of the interconnected identifiers or a range of preconfigured filters or using the powerful keyword search. The FAANG data is accessible by humans and machines with the persistent identifiers linking directly to the underlying data archives. The data is interoperable through use of open and widely accepted data formats, and all records are ensured to have met the high metadata standards that make extensive use of ontologies for standardising data descriptions. The Data Portal ensures data is reusable by associating mandatory detailed user submitted sampling, sequencing, and analysis protocols to each submitted data record. This provides highly detailed information on each study's methodology, and in even greater detail than the already rich metadata records. A specific protocol browsing page^6^ also assists the community in designing future experiments and further standardising how FAANG data is generated for future compatibility of comparative studies.

The FAANG data reuse policy is clearly associated with all FAANG records, and the prepublication data policy is supported through the clear labelling of data within the portal that has been published and thus is free of constraint for further research use. The automated association of publications with data records is particularly important for users to know what records are free from publication restriction in accordance with FAANG's data use policy^7^. This prohibits publication with obtained datasets until the data owners themselves have first published. To aid this, the Data Portal clearly displays with a green tick all datasets that have an associated publication (Figure 2). All of the codes for the FAANG Data Portal, data processing, and brokering are freely available under an Apache 2.0 licence^8^.

### Future Developments

The FAANG project is now moving into its next major phase, with a greater focus on harnessing functional ‘omic data from larger populations and leveraging recent technological advances such as single-cell ‘omics, species and tissue-cell atlases, pangenomes, and novel phenotypic prediction models. Collectively, these will further improve animal genome genotype-to-phenotype annotation and its translation to industrial applications to improve animal production. The Data Portal will continue to evolve alongside the communities' research priorities, and the DCC will develop new infrastructure and site features to effectively deliver and standardise these new data types. For agricultural single-cell ‘omics and cell atlases, the DCC will take advantage of the significant prior developments of the ENCODE consortium (Davis et al., 2018) and Human Cell Atlas projects (Regev et al., 2017).

The DCC and the wider FAANG bioinformatics community are focussed on ensuring open reproducible science. There is significant ongoing community effort to create reproducible analysis pipelines. To support this, the Data Portal is already preparing a functionality to link each analysis file to the reusable pipeline that produced it. A new browser page would also create a catalogue of standardised containerised FAANG pipelines to users for further downstream analysis. The Data Portal will look to develop links, wrappers, and infrastructure to enable rapid launching of cloud-based analysis through a range of providers. Discussions are ongoing to support mirroring of FAANG datasets and host the FAANG community's curated bioinformatics pipelines. Alongside the technical infrastructure and standardised pipelines, there is a need to train the current and next generation of scientists to effectively implement them. The FAANG Data Portal could release an online training resource that collates documentation, videos, and webinars on FAANG analysis methods and protocols. This will host resources produced by FAANG projects and links to training upcoming courses. This training, and the distributed data and analysis infrastructure, will be crucial for the successful application of functional data to farmed animal breeding programmes.

The current implementation of search within the data portal is based on inclusive keyword ranking. It is powerful for simple searches to get a view across the different FAANG data types but lacks the desired specificity when multiple search terms are provided. Currently specific multi-term searches need to be performed using the preconfigured filters on the data exploration tables (Figure 3) or using the API. To address this, an advanced query language search will be developed that provides a similar search customisation power to that available to programmatic users in the API. The new query language search will allow the user to search by multiple terms linked to specific fields to accurately narrow the returned results to the desired data records. This will mean that a search for “sex = female” and “species = sus scrofa” will only returning female pig records, rather than the current “female” and/or “sus scrofa” search that would return any female or any pig records. This will complement the existing API and data portal table filter searches that already make multiple-field/value search possible.

Automated and standardised visualisation of data across species, systems, tissues, and cell types will also be a key focal area. This includes both automated DCC-generated and user-provided visualisations. FAANG comparative and phenotypic datasets continue to increase in complexity, driving a need to build open-source systems to interrogate and visualise them for research and industrial applications. The Data Portal will also support improved cross referencing and linking to established biorepositories, breeding resources, and key data resources of phenotypic, climate, and functional data. Through its continued key role for FAANG, the Data Portal will continue to support high-quality functional annotation of animal genomes through open sharing of data complete with FAIR standardised rich metadata, and new portal features to support new technological developments for continued improvement in functional annotation of farmed and companion animal genomes.

## Project Links

The FAANG Data Portal—https://data.faang.org/

The FAANG Data Portal frontend code—https://github.com/FAANG/dcc-portal-frontend

The FAANG Data Portal validation and file conversion code—https://github.com/FAANG/dcc-validate-metadata

The FAANG metadata raw files—https://github.com/FAANG/dcc-metadata

The FAANG Data Portal documentation—https://dcc-documentation.readthedocs.io/en/latest/faq/.

## Data Availability Statement

Publicly available datasets were analysed in this study. This data can be found at: https://data.faang.org/home.

## Author Contributions

PH authored the paper. PH, AN, AS, and JF developed the Data Portal. PH, DZ, GC, and PF managed the project. All authors read and approved the final manuscript.

## Conflict of Interest

The authors declare that the research was conducted in the absence of any commercial or financial relationships that could be construed as a potential conflict of interest.

## Footnotes

^1^https://data.faang.org

^2^https://www.embassycloud.org/

^3^https://trackhubregistry.org/

^4^https://dcc-documentation.readthedocs.io/en/latest/faq/

^5^https://github.com/FAANG/comm-data-portal-projects/tree/master/projects

^6^https://data.faang.org/protocol/samples

^7^https://www.faang.org/data-share-principle

^8^https://github.com/FAANG/dcc-portal-frontend/blob/master/LICENSE
